# Epstein-Barr Virus (EBV)-Positive Diffuse Large B-Cell Lymphoma in a Young Patient: A Difficult Diagnosis

**DOI:** 10.7759/cureus.86254

**Published:** 2025-06-17

**Authors:** Motona Kumagai, Miyako Shimasaki, Akihiro Shioya, Sohsuke Yamada

**Affiliations:** 1 Department of Pathology II, Kanazawa Medical University, Uchinada, JPN; 2 Department of Pathology and Laboratory Medicine, Kanazawa Medical University, Uchinada, JPN

**Keywords:** c-myc, ebv, ebv-associated lymphoma, ebv-positive diffuse large b-cell lymphoma, immune escape, programmed death ligand 1 (pd-l1)

## Abstract

Epstein-Barr virus (EBV)-positive diffuse large B-cell lymphoma (EBV+DLBCL) was initially hypothesized to occur against a background of age-related immunodeficiency and was classified as EBV-positive DLBCL of the elderly. Subsequent reports of younger patients led to the renaming of the disease. A 17-year-old girl with no significant medical history presented with a left neck mass. Positron emission tomography-CT revealed augmented FDG accumulation in the left neck and multiple FDG accumulations in other lymph nodes, necessitating a biopsy. Histological findings revealed destruction of the follicular structure within the lymph nodes, with some parts exhibiting nodular formation due to fibrosis. Large Hodgkin/Reed-Sternberg (HRS)-like cells intermingled with small lymphocytes and histiocytes were observed. Immunostaining revealed that the HRS-like cells were positive for CD20 and EBER-ISH and negative for CD30 and CD15. The expression of PD-L1 was observed in tumor cells, whereas the cells were mostly negative for c-Myc. The patient was young and exhibited no symptoms indicative of immunodeficiency, thereby supporting the hypothesis of a pathogenesis derived from immune evasion. We focused on the relationship between PD-L1, which is involved in immune evasion mechanisms, and c-Myc, which may directly contribute to the immune escape of tumor B cells.

## Introduction

Epstein-Barr virus (EBV)-positive diffuse large B-cell lymphoma (EBV+DLBCL) was initially thought to occur against a background of age-related immunodeficiency and was classified as EBV-positive DLBCL of the elderly. Later studies identified cases occurring at a younger age, and the disease name was changed in the World Health Organization classification, revised 4th edition [1]. According to the most recent publication by the World Health Organization (WHO) 5th edition, this disease retains its designated nomenclature [2].

Patients with Epstein-Barr virus-positive (EBV+) large B-cell lymphoma in young patients without immunodeficiency are rare. A comprehensive analysis of 46 cases of young EBV+DLBCL patients (patients ≤45 years of age) with clinical and pathological features was conducted, which demonstrated that the patient population was predominantly male, exhibiting symptoms of lymphadenopathy, and 11% also had extranodal involvement [3]. Morphological analysis revealed the prevalence of T-cell/histiocyte-rich large B-cell lymphoma-like patterns [3]. Tumor cells expressed B-cell antigens, were frequently CD30 and PD-L1 positive, and exhibited a nongerminal center immunophenotype [3]. Patients with younger-onset EBV+DLBCL demonstrated a notably higher overall survival rate compared to their older counterparts with the same diagnosis [3].

Age-related immunodeficiency is believed to be associated with tumorigenesis in diffuse large B-cell lymphoma cells and classical Hodgkin lymphoma and exhibits a high rate of programmed death-ligand 1 (PD-L1) expression in tumor cells, indicating that an immune escape mechanism may play a significant role in its development [4,5].

Programmed cell death receptor 1 (PD-1) and PD-L1 are important immune checkpoint molecules that play a key role in the T cell-mediated immune response, regulating tumor immune escape [6-8].

In addition, c-Myc is one of the most essential transcription factors, regulating diverse cellular functions including proliferation, growth, and apoptosis. Dysregulation of c-Myc is crucial in the development of several B-cell lymphomas [9].

We focused on the relationship between PD-L1, which is involved in immune evasion mechanisms, and c-Myc, which may directly contribute to the immune escape of tumor B cells.

In this report, we present a case of EBV + DLBCL that posed significant diagnostic challenges owing to the presence of fibrous septa that bore a striking resemblance to nodular sclerosis classical Hodgkin lymphoma (NSCHL) and scattered large Hodgkin/Reed-Sternberg (HRS) cell-like atypical cells.

## Case presentation

The patient was a 17-year-old girl. Her chief complaint was a mass on the left side of the neck. Her family history and medical history were unremarkable. The patient noted the presence of a left cervical mass approximately one month prior; notably, B symptoms such as fever were absent in this case. Echography revealed a seven-centimeter lymphadenopathy in the neck, which was suspected to be malignant lymphoma. Consequently, a lymph node biopsy was performed.

**Figure 1:**
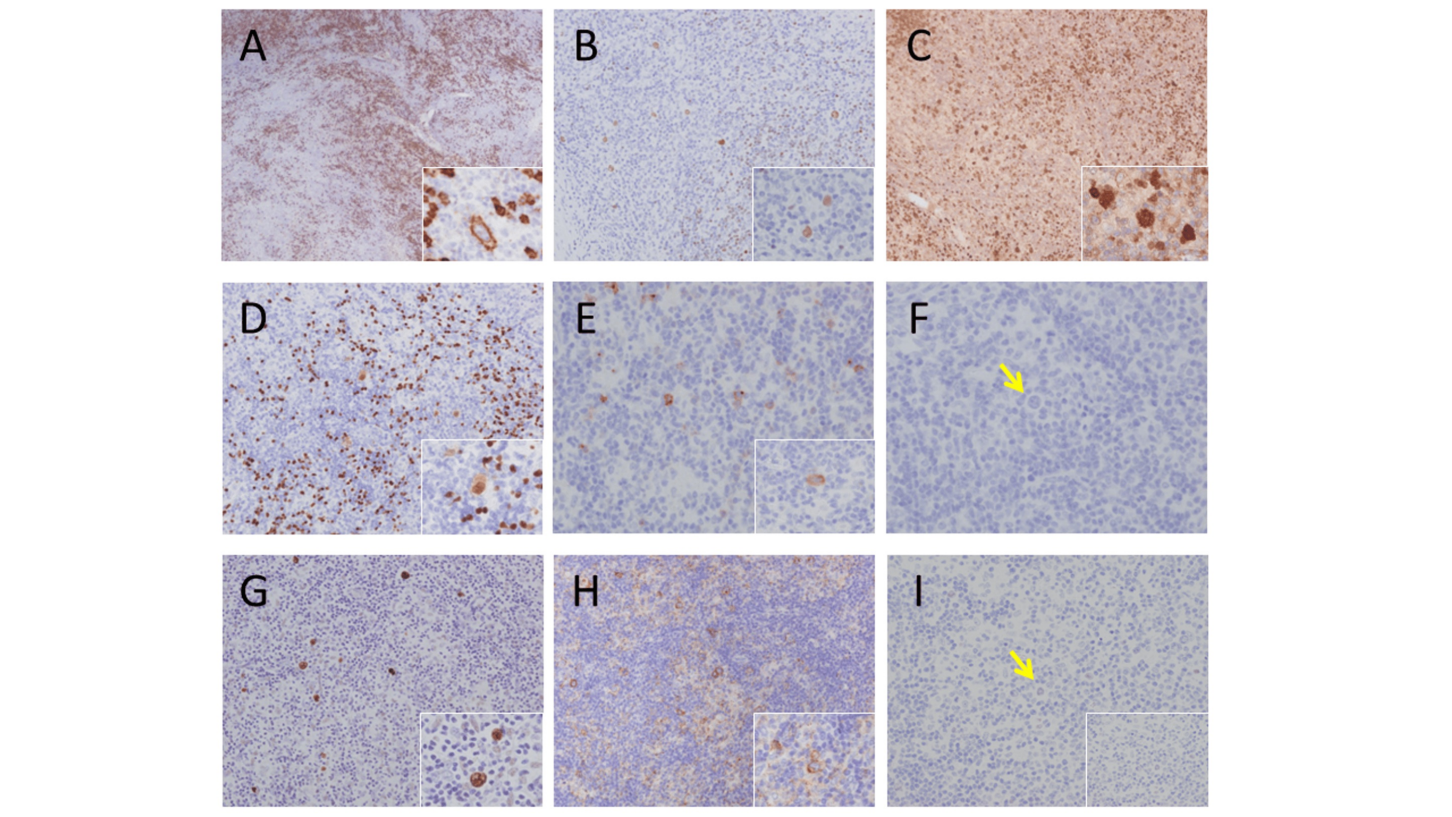
Immunohistological findings A: Immunostaining for CD20. Large cells are positive; B: Immunostaining for Oct-2. Positive for large cells; C: Immunostaining for BOB-1. Positive for large cells; D: Immunostaining for PAX5. Weakly positive for large cells; E: Immunostaining for CD30. Some large cells are positive; F: Immunostaining for CD15. Negative for large cells. The yellow arrows point to tumor cells; G: Immunostaining for EBER. Positive for large cells; H: Immunostaining for PD-L1. Positive for large cells; I: Immunostaining for c-myc. Most large cells are negative. The yellow arrows point to tumor cells.

Immunohistochemically, the large atypical cells were positive for CD20, CD79a, Oct-2, and BOB-1; Pax-5 showed a pale positive image compared to surrounding B cells; CD30 was partially positive; CD15, STAT6, and pSTAT6 were negative, positive for EBER-ISH, positive for PD- L1 (about 70%), and mostly negative for c-Myc (Figures 1A-1I).

Small cells were mainly CD3-, CD5-, and CD7-positive, with CD4- and CD8-positive cells being equally positive. The results of immunostaining of large cells are summarized in Table 1.

**Table 1:** Summary of immunohistochemical findings of large cells

Antiobody	Large cells
CD20	＋
CD79α	＋
PAX5	＋
Oct-2	＋
BOB-1	＋
bcl-2	ー
bcl-6	(weak)+
CD10	ー
MUM-1	＋
CD30	(partial)+
CD15	ー
STAT6	ー
pSTAT6	ー
CD3	ー
CD5	ー
CD4	ー
CD8	ー
CD7	ー
Granzyme B	ー
perforin	ー
EBER-ISH	＋
PD-L1	＋
c-Myc	(partial)ー

Based on the histological findings, we initially considered tuberous sclerosis to be classic Hodgkin lymphoma (CHL). However, immunohistochemistry showed that the HRS-like cells were only partially positive for CD30. In CHL, the expression of B cell markers is usually reduced or absent; however, in this case, although the expression of PAX5 was somewhat weak, CD20, CD79a, PAX5, Oct2, and BOB1 were all positive. Furthermore, CD15, STAT6, and pSTAT6, which are highly positive in CHL, were negative. Based on these findings, the diagnosis was EBV+ DLBCL in a young patient, not CHL.

## Discussion

Nicolae et al. identified EBV-positive DLBCL in three morphological patterns. Three morphological patterns were identified in EBV+ DLBCL: (i) T-cell/histiocyte-rich large cell lymphoma-like (THRLBCL-like) (78%); (ii) grey zone lymphoma (GZL) (15%); and (iii) diffuse large B-cell lymphoma, not otherwise specified (DLBCL, NOS) (6.5%). Tumor cells expressing B-cell antigens are frequently positive for CD30 and PD-L1 and exhibit an activated B-cell-like/non-germinal center type [3].

In the most common histology, T-cell/histiocyte-rich large cell lymphoma-like (THRLBCL-like) cases, lymphoid follicles are largely absent and EBV-positive large cells are scattered in an abundant inflammatory cell background consisting primarily of histiocytes and small lymphocytes. In some cases, a fibrous capsule or a partially fibrous septum is observed.

Neoplastic B cells are large, encompassing HRS-like cells and immunoblast-like cells. Tumor cells are frequently observed in sporadic configurations as either solitary or loose clusters. In certain instances, these cells manifest in a sheet-like configuration. Additionally, the presence of plasma cells and eosinophils among the infiltrating inflammatory cells is observed.

Morphologically, THRLBCL and NSCHL are different diseases, although they can be differentiated from this disease by immunostaining.

In THRLBCL, HRS-like cells are negative for EBER-ISH and can be differentiated from this disease. In CHL, including NSCHL, HRS-like cells are usually positive for CD15 and rarely express both Oct2 and BOB1, and many are negative for both or either. In this disease, CD15 is negative in most cases and both Oct2 and BOB1 are positive.

In this case, epithelioid cells were prominent; however, they were considered to correspond to a diagnosis of THRLBCL-like EBV+ DLBCL.

Recent studies have demonstrated that EBV+DLBCL occurs in a wide range of age groups. In addition, a growing body of literature clearly demonstrates differences in the clinical and pathological features between EBV+DLBCL in the elderly (DLBCL-E) and EBV+DLBCL in younger patients (DLBCL-Y) [3,10]. In an analysis of DLBCL-Y patients (< 45 years of age without immunodeficiency), the male-to-female ratio was 3.6:1, with a median age of 23 years (age 4-45 years). All patients presented with lymphadenopathy and 11% had extranodal involvement. In contrast, 70-80% of patients with DLBCL-E had extranodal involvement. DLBCL-Y exhibits reduced extranodal involvement and has a more favorable prognosis than DLBCL-E [4]. These observations indicate that DLBCL-Y and DLBCL-E represent distinct clinical entities, suggesting the potential for divergent pathogenesis of both lymphomas.

Age-related immunodeficiency is believed to be associated with tumorigenesis in DLBCL-E and DLBCL-Y cells, similar to CHL, and exhibits a high rate of PD-L1 expression in tumor cells, indicating that an immune escape mechanism may play a significant role in its development [4,5].

PD-L1, also known as CD274 or B7H1, is expressed on tumor cells and plays an important role in tumor immune escape and the formation of a permissive immune microenvironment through various mechanisms. First, it binds to its receptor PD-1, thereby inhibiting its activity and either permitting tumor immune evasion or inducing anergy in T cells, which suppresses anti-tumor responses. Second, it renders tumor cells resistant to CD8+ T cells and Fas ligand-mediated lysis. Thirdly, it tolerates T cells by reverse signaling through T cell-expressed CD80 [11-13].

The application of immunostaining for PD-L1 as a means to ascertain the presence of immune evasion mechanisms in tumor cells enables the classification of EBV+DLBCL and B cell lymphoproliferative disorder into three distinct categories: immunodeficient, immune evasion, and immunodeficient + immune evasion. The immunodeficient category, characterized by PD-L1 negativity, is believed to result from immunodeficiency induced by aging or methotrexate utilization. The immune escape type is PD-L1-positive and is less frequently associated with immunodeficiency. The immunodeficiency + immune escape type is PD-L1-positive and is thought to be associated with immunodeficiency [14].

In this case, the expression of PD-L1 was observed in the tumor cells. The patient was a teenager with no previous medical history and no symptoms suggestive of immunodeficiency, thus indicating that the development of the disease was due to immune evasion.

Recent studies indicate that Myc not only contributes to tumorigenesis by its effects on cell proliferation and differentiation but also plays an important role in promoting escape from anti-tumor immune responses [15].

c-Myc has been identified as the master transcription factor of EBV latent III proliferative B cells, and its aberrant expression has been linked to immune evasion in tumor B cells [16]. c-Myc reduces the expression of active immunomodulatory surface molecules, such as PD-L1. In addition, previous studies have demonstrated that suppression of the expression of c-Myc in vitro results in the increased expression of B7-H1 (PD-L1) mRNA and that the suppression of c-Myc increases the surface membrane expression of B7-H1 (PD-L1) [17]. Therefore, we hypothesized that the increased PD-L1 expression in EBV-infected B cells results from the suppression of c-Myc.

Immunostaining for c-Myc was negative in the majority of tumor cells. Consequently, it is proposed that the repression of the c-Myc protein expression may be a contributing factor to the augmented PD-L1 expression in tumor cells in this particular instance. The presence of PD-L1 on tumor cells, in conjunction with elevated PD-L1 expression within the tumor microenvironment, is likely to facilitate immune evasion. However, in the patient cohort examined by Nicolae et al., PD-L1 expression did not appear to result in adverse outcomes [3].

## Conclusions

It is hypothesized that EBV+DLBCL in young individuals frequently expresses PD-L1 and that immune evasion mechanisms are deeply involved in its development. The suggestion that increased PD-L1 expression in EBV-infected B cells is due to c-Myc suppression could lead to tumor B cells evading the immune system.

This case occurred in a young woman who was inherently healthy, and no symptoms suggesting immunodeficiency were observed, supporting the notion that the tumor developed via immune evasion. In fact, tumor cells exhibited PD-L1 expression. Immunostaining for c-Myc was performed, and most tumor cells were negative. Therefore, it is suggested that the suppression of c-Myc protein expression may be involved in the increased PD-L1 expression observed in the tumor cells of this case.
